# Urinary tract infection stewardship: A urinary antibiogram and electronic medical record alert nudging narrower-spectrum antibiotics for urinary tract infections

**DOI:** 10.1017/ash.2021.163

**Published:** 2021-06-29

**Authors:** Maryrose Laguio-Vila, Mary L. Staicu, Mary Lourdes Brundige, Jose Alcantara, Hongmei Yang, Ebbing Lautenbach, Ghinwa Dumyati

**Affiliations:** 1 Department of Infectious Diseases, Rochester General Hospital, Rochester, New York; 2 Department of Pharmacy, Rochester General Hospital, Rochester, New York; 3 Department of Internal Medicine, Rochester General Hospital, Rochester, New York; 4 Departments of Biostatistics and Computational Biology, University of Rochester Medical Center, Rochester, New York; 5 Department of Infectious Diseases, University of Pennsylvania, Philadelphia, Pennsylvania; 6 Department of Infectious Diseases, University of Rochester Medical Center, Rochester, New York

## Abstract

An antimicrobial stewardship intervention consisting of a urinary antibiogram and an electronic health record best-practice advisory promoted narrower-spectrum antibiotics for uncomplicated urinary tract infections in hospitalized patients. Over 20 months, the intervention significantly reduced ceftriaxone orders by 48% (*P* < .001) and increased cefazolin use 19 times from baseline (*P* < .001).

Urinary tract infections (UTIs) are the second most common indication for inpatient antibiotics in the United States,^1^ making them a prime target for antimicrobial stewardship programs (ASPs). The 2010 Infectious Diseases Society of America guidelines for acute uncomplicated cystitis recommend narrow-spectrum antibiotics (NSAs),^2^ yet many hospitalized patients receive broad-spectrum antibiotics (BSAs).^1^ To reduce BSA use, such as third-generation cephalosporins, and to promote first-generation cephalosporin use for uncomplicated UTIs, we developed a urinary antibiogram and an electronic health record (EHR) best-practice advisory (BPA). We then measured their impact on antibiotic prescriptions for UTIs.

## Methods

This single-center, quasi-experimental study was conducted in a 528-bed tertiary-care hospital in Rochester, New York. All antibiotics are ordered by computerized physician order entry in EHR software (Epic, Verona, WI) that includes a mandatory field for infectious indication. “Urinary tract infection” is 1 of 18 fields, encompassing both cystitis and pyelonephritis, regardless of infection severity. The ASP team prospectively receives 2 automated reports monthly: (1) antibiotic orders subcategorized by indication, and (2) days of therapy (DOT) without indication stratification. Support was provided by the Centers for Disease Control and Prevention Emerging Infection Program. The Rochester General Hospital Institutional Review Board approved this study and waived informed consent.

### Intervention

The intervention involved 3 cephalosporins (ceftriaxone, cefazolin, and cephalexin) over 3 phases. From January 2016 through January 2018 (phase 0), monthly DOT and orders with UTI indication were prospectively calculated per 1,000 patient days. In collaboration with the microbiology laboratory, an inpatient urinary antibiogram of the most common urine pathogens of 2017 was created: *Escherichia coli*, *Klebsiella pneumoniae*, *Proteus mirabilis*, and *Enterococcus* spp. The laboratory introduced routine cefazolin Kirby-Bauer disc diffusion assays on urinary isolates for accurate susceptibility testing and release for clinical use (CLSI supplement M100, Wayne, PA). From February through July 2018 (phase 1), the ASP team gave lectures on the antibiogram and distributed a pocket reference card (Supplementary Material online). The ASP team also encouraged the application of the systemic inflammatory response syndrome (SIRS) criteria to identify potentially uncomplicated UTIs. Cases meeting 0–1 SIRS criteria were suggested to be low risk for negative outcomes and eligible for empiric NSA. Daily prospective audit of all new ceftriaxone orders identified additional cases eligible for NSA. In phase 2 (August 2018–October 2019), an EHR BPA designed by ASP with health informatics was introduced (Supplementary Material online). This BPA pop-up window interrupted all providers ordering ceftriaxone with the UTI indication, explaining that ceftriaxone was a BSA and suggesting the use of NSAs according to the antibiogram. The primary outcome was changes in UTI treatments, measured as the number of ceftriaxone, cefazolin, and cephalexin orders for UTI per 1,000 patient days. Secondary outcomes were changes in each cephalosporin’s total DOT per 1,000 patient days.

### Analysis

Negative binomial regression for data with overdispersion was applied to estimate and compare monthly cephalosporin-specific UTI orders and DOTs per 1,000 patient days among different study phases. We report rate ratios (RRs) and the corresponding 95% confidence intervals (CIs) after the interventions versus before the interventions. Interrupted time series (ITS) analysis by segmented regression models was used to assess the extent to which the interventions affected levels and trends in the rates of antibiotic UTI orders and DOT over the 3 phases of the intervention. The Durbin-Watson test was used to assess autocorrelation. Except where specified, all reported results compare phase 2 with phase 0. Analyses were conducted using SAS version 9.4 software (SAS Institute, Cary, NC).

## Results

Rate ratios by negative binomial regression showed significant decreases in the rate of ceftriaxone UTI orders by Phase 2 (RR, 0.52; 95% CI, 0.48–0.55; *P* < .001) and in the rate of total ceftriaxone orders (RR, 0.87; 95% CI, 0.83–0.91; *P* < .001) (Table 1a). Conversely, there were significant increases in the rate of cefazolin UTI orders (RR, 19.59; 95% CI, 14.87–25.81; *P* < .001) and the rate of cefazolin DOT (RR, 1.13; 95% CI, 1.09–1.17; *P < .*001). We detected significant increases in the rates of cephalexin UTI orders (RR, 2.43; 95% CI, 2.11–2.79, *P* < .001), cephalexin total orders (RR, 1.18; 95% CI, 1.08–1.30; *P* < .001), and cephalexin DOT (RR, 1.32; 95% CI, 1.21–1.44; *P* < .001).

**Table 1a.** Antibiotic Use Rate Ratios by Urinary Tract Infection (UTI) Antibiotic Orders and Total Antibiotic Days of Therapy by Phase of Intervention

Antimicrobial Agent	Phase 0		Phase 1						Phase 2					
	Orders/Patient Days	Rate^a^	Orders/Patient Days	Rate^a^	RR	95% LCL	95% UCL	Pr > |z|^b^	Orders/Patient Days	Rate^a^	RR	95% LCL	95% UCL	Pr > |z|^b^
UTI Antibiotic Orders														
Ceftriaxone	6,944/621,746	11.17	1,172/121,363	9.81	0.88	0.79	0.97	**.011**	2,705/449,173	5.76	0.52	0.48	0.55	**<.001**
Cefazolin	102/621,746	0.16	161/121,363	1.32	8.07	5.61	11.61	**<.001**	1,395/449,173	3.21	19.59	14.87	25.81	**<.001**
Cephalexin	857/621,746	1.38	276/121,363	2.35	1.71	1.39	2.1	**<.001**	1,487/449,173	3.34	2.43	2.11	2.79	**<.001**
Total Antibiotic Days of Therapy														
Ceftriaxone	29,983/621,746	48.17	6,669/121,363	45.53	0.95	0.86	1.04	.238	21,159/449,173	47.46	0.99	0.92	1.05	0.651
Cefazolin	47,177/621,746	75.86	11,385/121,363	77.63	1.02	0.97	1.08	.38	38,203/449,173	85.62	1.13	1.09	1.17	**<.001**
Cephalexin	7,223/621,746	11.62	1,847/121,363	12.58	1.08	0.96	1.22	.21	6,819/449,173	15.35	1.32	1.21	1.44	**<.001**

Note. RR, rate ratio; LCL, lower confidence limit; UCL, upper confidence limit.

a Rate estimated with negative binomial regression.

b *P* value for the *z* statistic. Bold indicates statistical significance.

**Fig. 1.**
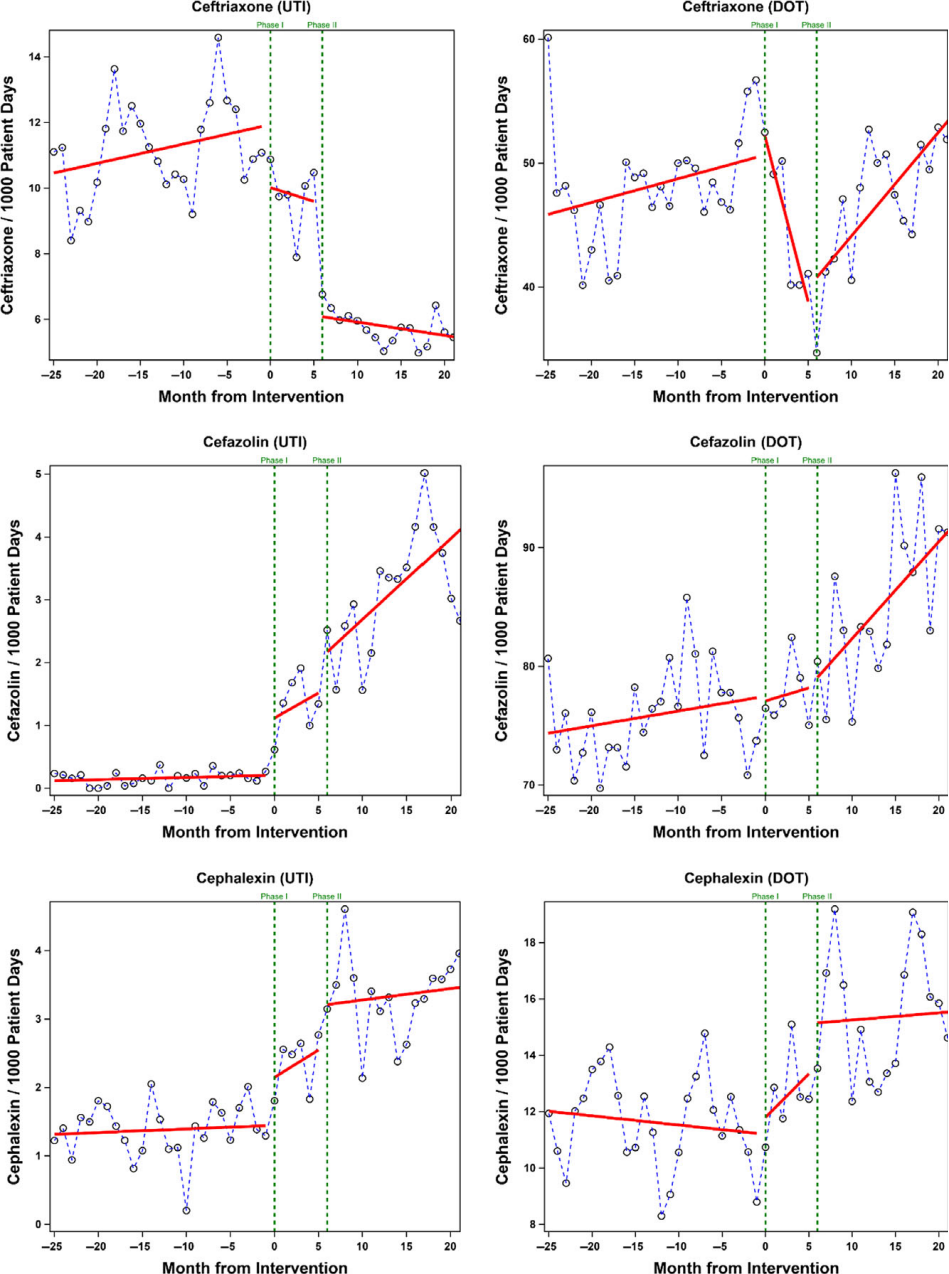
Changes in urinary tract infection (UTI) antibiotic orders (left) and total days of therapy (DOT) per 1,000 patient days (right) associated with implementation of a urinary antibiogram (phase 1) and an electronic medical record best practice advisory (phase 2).

By ITS, a significant decrease in the levels of ceftriaxone UTI orders (level change, 5.61; *P =* .001), total orders (level change, −9.38; *P* < .001), and DOT (level change, −14.87; *P* < .001) occurred from phase 0 to phase 2 (Fig. 1 and Table 1b). A significant increase in the level of cefazolin UTI orders also occurred without changes in the levels of cefazolin total orders or DOT. Significant increases in the levels of cephalexin UTI orders and DOT also occurred. The results of the Durbin-Watson test suggested no indication for autocorrelation in the study data, which was confirmed by residual plots.

**Table 1b.** Changes in Levels and Trends in Antibiotic Use per 1,000 Patient Days by Segmented Regression Analysis of Interrupted Time Series From Phase 0 to Phase 2

Antimicrobial Agent		Change in Level	Standard Error	Pr > |t|^a^	Level Change From Phase 0, %	Change in Trend	Standard Error	Pr > |t|^a^
Ceftriaxone	UTI orders	−5.61	0.97	**<.001**	−47	−0.10	0.07	.133
	Total orders	−9.38	2.02	**<.001**	−24	−0.12	0.14	.370
	DOT	−14.87	3.48	**<.001**	−29	0.64	0.23	**.009**
Cefazolin	UTI orders	1.19	0.41	**.006**	570	0.13	0.03	**<.001**
	Total orders	−11.01	6.19	.083	−8	−0.67	0.42	.114
	DOT	−3.31	3.59	.363	−4	0.69	0.24	**.007**
Cephalexin	UTI orders	1.66	0.41	**<.001**	115	0.01	0.03	.680
	Total orders	1.47	1.32	.272	17	0.06	0.09	.489
	DOT	3.80	1.61	**.023**	34	0.06	0.11	.595

Note. UTI, urinary tract infection; DOT, days of therapy.

a *P* value for the *t* test. Bold indicates statistical significance.

## Discussion

This intervention significantly reduced ceftriaxone ordering by 48% while increasing cefazolin and cephalexin prescribing by 19 times and 2.4 times over the baseline, respectively. Previously, cefazolin was not commonly prescribed for UTIs of any severity in our institution. Thus, collaboration with the microbiology laboratory was integral to creating a urinary antibiogram demonstrating high local NSA susceptibility to facilitate provider buy-in. The use of institution-specific syndromic antibiograms may provide more accurate estimates of resistance and greater potential to better guide empiric treatment.^3,4^ Fortunately, the local cefazolin susceptibility of 92%–95% for common urinary pathogens made it an ideal empiric agent. The ASP team considered promoting trimethoprim-sulfamethoxazole and nitrofurantoin to remain consistent with national guidelines^2^; however, local prescriber concerns for low potency and adverse events, and practices preferring intravenous antibiotics for hospitalized patients precluded this step. Local ASPs and their associated laboratories could explore other NSAs and tailor recommendations accordingly.

ASP teams can leverage the customizability of the EHR to efficiently target prescribers at optimal times and with specific information to guide clinical decision making.^5^ This BPA was designed to reach providers at the final moment of ceftriaxone prescribing and to reinforce ASP principles. We observed that education in phase 1 significantly influenced cefazolin orders but with minimal changes in ceftriaxone orders. However, significant changes in ceftriaxone ordering materialized in phase 2, likely due to the BPA.

The impact on total NSA DOT was modest, with no significant change in total ceftriaxone DOT. This finding was expected because UTIs represent only a portion of infectious indications. Prior to this intervention, >95% of cefazolin orders were for surgical prophylaxis, diluting any subsequent changes in cefazolin metrics. Similarly, ceftriaxone DOT was likely confounded by the concomitant national ampicillin-sulbactam shortage, which diverted many regimens to include ceftriaxone.

Sociobehavioral studies suggest that stewardship interventions that do not interfere with workflow or negatively affect efficiency may be more readily accepted.^6^ In this intervention, we deliberately incorporated the widely used SIRS criteria into antibiotic decision making. The intent was to bolster prescriber confidence that each case with low SIRS criteria was low risk for negative clinical outcomes should an NSA provide inadequate bacterial coverage. Secondarily, it cognitively links the intervention to a process embedded in provider workflow. We are unaware of other studies using SIRS criteria in a stewardship strategy.

This study has several limitations. The high susceptibility of urinary pathogens to these NSA may not be widely applicable. However, collaboration with microbiology to identify local NSAs could be replicated. Second, because the BPA is UTI specific, providers determined to order ceftriaxone potentially could achieve a “workaround” by choosing non-UTI indications, leading to falsely low measurement of ceftriaxone UTI orders. Last, the study did not assess clinical outcomes such as NSA effectiveness and safety. This aspect will be a focus of future investigations.

In conclusion, a multimodal syndromic stewardship intervention introducing a urinary antibiogram and a BPA significantly reduced ceftriaxone and successfully increased NSAs for UTI treatments in hospitalized patients.

## Supplementary material

For supplementary material accompanying this paper visit https://doi.org/10.1017/ash.2021.163. click here to view supplementary material
