# Supplementary material for: Urinary tract infection stewardship: A urinary antibiogram and electronic medical record alert nudging narrower-spectrum antibiotics for urinary tract infections
Source: Antimicrob Steward Healthc Epidemiol. 2021 Jun 29;1(1):e8. doi: 10.1017/ash.2021.163 (PMC9495411; doi:10.1017/ash.2021.163)

**Supplemental material**

**Figure**. Pocket card with urinary antibiogram and antibiotic recommendations, stratified by systemic inflammatory response syndrome (SIRS) criteria (left). Electronic medical record Best Practice Advisory pop-up window recommending narrow-spectrum antibiotics when ceftriaxone with the indication of a urinary tract infection is ordered (right).


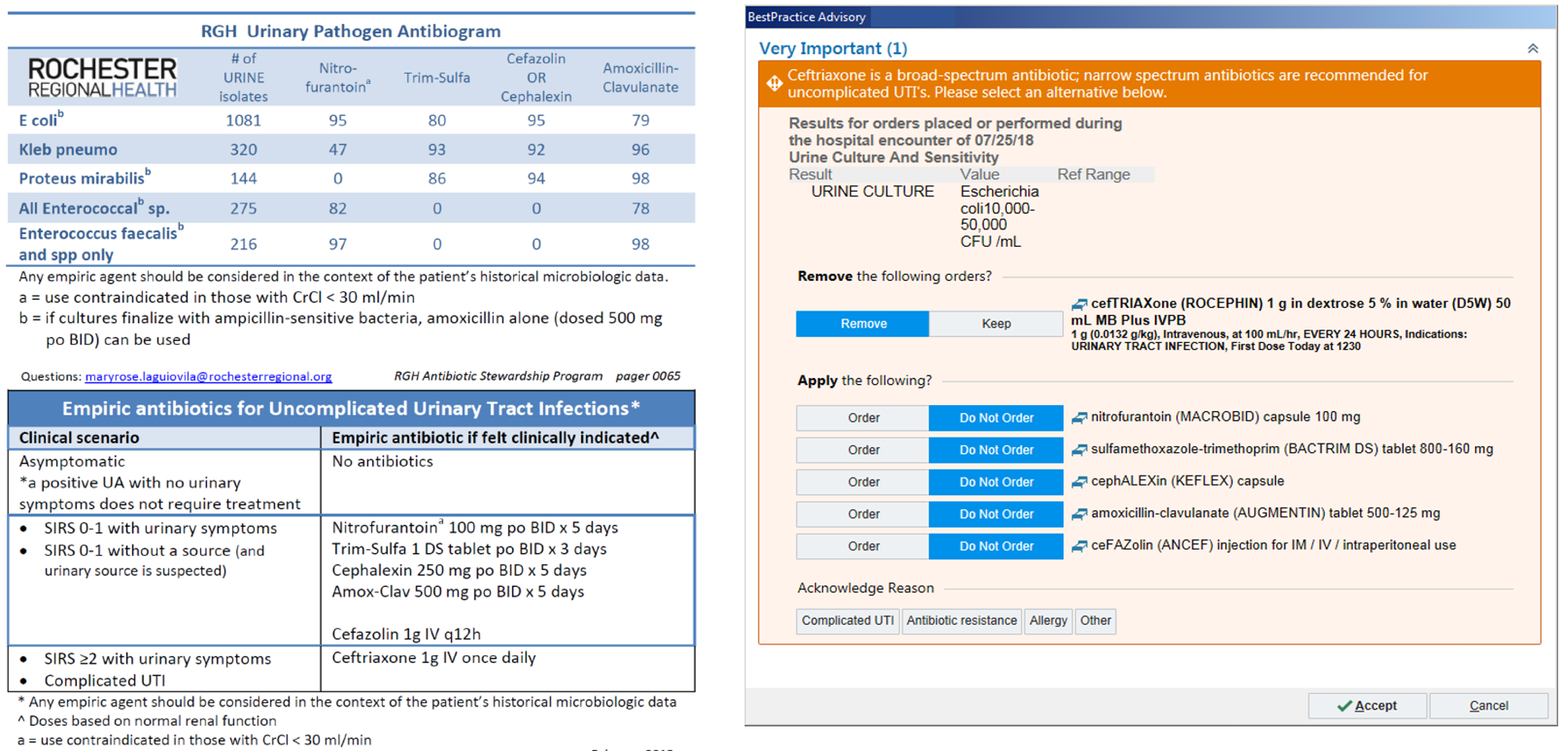

Supplement: Supplementary file 1 [file S2732494X21001637sup001.docx]
